# From hybridization theory to microarray data analysis: performance evaluation

**DOI:** 10.1186/1471-2105-12-464

**Published:** 2011-12-02

**Authors:** Fabrice Berger, Enrico Carlon

**Affiliations:** 1 Institute for Theoretical Physics, KULeuven, Celestijnenlaan 200D, B-3001 Leuven, Belgium

## Abstract

**Background:**

Several preprocessing methods are available for the analysis of Affymetrix Genechips arrays. The most popular algorithms analyze the measured fluorescence intensities with statistical methods. Here we focus on a novel algorithm, AffyILM, available from Bioconductor, which relies on inputs from hybridization thermodynamics and uses an extended Langmuir isotherm model to compute transcript concentrations. These concentrations are then employed in the statistical analysis. We compared the performance of AffyILM and other traditional methods both in the old and in the newest generation of GeneChips.

**Results:**

Tissue mixture and Latin Square datasets (provided by Affymetrix) were used to assess the performances of the differential expression analysis depending on the preprocessing strategy. A correlation analysis conducted on the tissue mixture data reveals that the median-polish algorithm allows to best summarize AffyILM concentrations computed at the probe-level. Those correlation results are equivalent to the best correlations observed using popular preprocessing methods relying on intensity values. The performances of each tested preprocessing algorithm were quantified using the Latin Square HG-U133A dataset, thanks to the comparison of differential analysis results with the list of spiked genes. The figures of merit generated illustrates that the performances associated to AffyILM(medianpolish), inferred from the present statistical analysis, are comparable to the best performing strategies previously reported.

**Conclusions:**

Converting probe intensities to estimates of target concentrations prior to the statistical analysis, AffyILM(medianpolish) is one of the best performing strategy currently available. Using hybridization theory, probe-level estimates of target concentrations should be identically distributed. In the future, a probe-level multivariate analysis of the concentrations should be compared to the univariate analysis of probe-set summarized expression data.

## Background

During the last decade, high-throughput technologies have been extensively used to monitor the expression profile of known and predicted genes. Those studies allowed to compare samples between healthy and pathologic tissues or cell-types, to monitor the effects of several drugs, and to describe sets of genes based on their implication in several biological processes [1-9]. As several microarray platforms are available (e.g. single color, dual color, high density arrays), different preprocessing and statistical analysis tools were developed, according to the specificities of each system. Here, we focus on the Affymetrix Genechip family products. The old generation chips used two types of probes to monitor the expression: Perfect Match probes (PM) and Mismatch Probes (MM), with the aim of characterizing non-specific binding thanks to the introduction of a mismatch in the central position of the probe. Irizarry *et al. *showed that the MM signal is also dependent on the PM target concentration, thus leading to a more complex situation where each MM probe signal should be interpreted using specific and non-specific binding [10,11]. The original idea of subtracting MM signal to PM signal before statistical analysis thus evolved, in different directions: several groups recommended to use PM-only signal, where others used an adjusted MM signal [10,11]. MM probes were also used as weak specific binders to estimate the background and saturation level [12]. Note that in its latest Genechips for expression arrays Affymetrix makes only use of PM probes, and completely redesigned probe sequences. In the newest chips, the probes are selected from the whole transcript and not from its 3'-end as it used to be the case. The non-specific binding is assumed to be common for all probes, and is assessed with a set of anti-genomic probes. Such novelties in the design call for a new critical evaluation of preprocessing methods, which is the aim of this paper.

When comparing several samples, algorithms need to take discrepancies between arrays into account, such as a slight difference in the amount of hybridized material (biological and technical replicates, precision of the pipetting device, hybridization time ...). The study of probe intensities thus traditionally implies a normalization step and/or the design of an appropriate statistical model. From a statistical point of view, several probes targeting the same transcript would ideally be jointly analyzed, using a multifactorial procedure - like the ANOVA-2 procedure - to be able to compare the expression of a transcript between two conditions (i.e. healthy/disease), and several samples (replicates). However, the probe-specific signal are not identically distributed, so that the iid requirement of ANOVA-2 procedures is not met. Discrepancies between PM probes targeting the same sequence have been reported by several authors. Depending on probe-specific sequences, several processes have been reported to explain the important biases observed in PM signals [13,14]. First, thanks to a more refined knowledge about transcript sequences and their locations in the genome, many probeset definition have been redefined and many probes have been redesigned in the successive generations of arrays. To correctly analyse old-generation arrays, several authors used alternative chip definition files and probe annotation tables [15-19]. Other sources of outlying effects have been reported by several authors. For instance, probes targeting distinct transcripts were found to be systematically correlated accross a large number of experiments, and fail to correlate with respect to the rest of the probes designed to target the same transcript (these are probes containing G-stacks, T7-primer sequences ...) [13,14]. Cross-hybridization effects may occur, as probe sequence may complement parts of the sequence from other transcripts. Listing all the factors that leads to outlying probe signals is not the purpose of this paper, but those examples illustrates the complexity of the models needed to analyse the data appropriately. Most preprocessing methods adress this issue by summarizing the probe-level signal into a unique probeset-level score, and this step is performed by excluding outliers or giving them a smaller weight as compared to other probes (Average Difference in MAS 4.0, 1-step Tukey-Biweight in MAS 5.0, Medianpolish in RMA and GC-RMA) [11,20-22].

The aim of this paper is to provide a general test of performance of some preprocessing methods for Genechips, focusing in particular on affyILM, an algorithm recently made available as a Bioconductor package and developed by us. This algorithm is based on physical modelling of the hybridization process and uses a generalized Langmuir isotherm [23] to estimate the concentration of transcripts from the raw data, using thermodynamics principles. The inputs are hybridization free energies obtained from experimental values which measure the affinity for each probe to bind to the complementary transcript. Several papers in the past decade discussed the physical modeling of the hybridization process in GeneChips [23-30]. AffyILM is fully based on underlying thermodynamics of hybridization and the basic principles were discussed in previous publications (see e.g. [31,32]). In this test of performance we considered old- and new-generation (i.e. PM only) chips and compared affyILM using several popular summarization methods (median, average difference, 1-step Tukey-Biweight, MBEI, medianpolish), with other preprocessing methods (MAS 5.0, RMA, GCRMA, Plier, FARMS,dChip) [11,20-22,33-35].

## Results and Discussion

Preprocessing microarray data is commonly performed on a four-step basis: background correction, pm/mm correction, normalization and summarization. Several algorithms have been reported to treat each of those steps, and this field of research has evolved in a combinatorial way, to define the best strategy [36]. To improve the understanding of the results described in this article, we have compared the performances with regards to MAS 5.0 and Plier (provided by Affymetrix), RMA and GCRMA, dCHIP and FARMS [11,20-22,33-35]. Those softwares have been the most widely used over ten years, and were studied in several performances studies [36-39].

Our aim is to provide a preprocessing algorithm based on physical chemistry. We used the extended Langmuir model, which is discussed in Ref. [23], to compute the physical concentration of the targets associated to each of the probes in the chip. Assuming that the differences between arrays are due mainly to the variable amount of sample which is hybridized, we performed a global multiplicative rescaling of the estimated concentrations, so that the pairwise comparison of concentrations is characterized by a slope equal to one between arrays.

The estimation of non specific binding implies platform-generation-specific features. The use of the Langmuir Isotherm to estimate concentrations is presented here, first ignoring this background-correction step, so that interpretation of the results does not depend on a difference between algorithms used with regard to the specificities related to each generation of array. As a consequence, the variability of probe-specific concentrations may be somewhat over-estimated (a constant background noise introduced in the Langmuir Isotherm may lead to a variable over-estimation of the concentration, as this part of the signal should not be associated to the probe-specific free energy). For this reason, the individual analysis reported here has been performed using both the Student t-test and variants of this test designed to stabilize the variance, at the probe-set level, using shrunken estimates of variability (regularized t-test and window t-test) [38,40].

### Correlation of differential expression analysis

To test the performance of affyILM, we selected the Affymetrix Tissue Mixture Study (available on Affymetrix's Website) [41]. This study involves two different genechips: the old HG-U133 Plus 2 and the new (PM-only) Hugene 1.0 ST array. Table 1 summarizes the design of the tissue mixture study: heart and brain samples were mixed in various ratios. Such a strategy allows to perform two types of comparisons: (i) Within-platform comparisons quantifies the robustness of the methods against biologival noise and (ii) between-platform comparisons quantifies the correlations between HG-U133Plus2 and Hugene 1.0 ST arrays. To quantify the performances of the differential expression analysis depending on the preprocessing strategy, we computed the correlation coefficient of Pearson, Kendall and Spearman on the log10 value of the p-values resulting from the differential expression tests that are compared. All three correlation coefficients provided similar results. In general, the Pearson correlation coefficient spanned the widest range of values, best highlighting the differences between the methods, and was therefore used in this paper.

**Table 1:** Description of the tissue mixture study

Sample Name	Brain (%)	Heart (%)
Mix 1	0	100
Mix 2	5	95
Mix 3	10	90
Mix 4	25	75
Mix 5 (a-c)	50	50
Mix 6	75	25
Mix 7	90	10
Mix 8	95	5
Mix 9	100	0

Each line refers to a mixture of brain and heart samples. For each mixture, the contribution of each sample (%) is given in the second and third columns. The mixture of equal amounts of brain and heart samples was conducted 3 times, leading to Mixes 5 a,b and c. Each mixture was then hybridized on 3 HG-U133Plus2 arrays and 3 HuGene 1.0 ST arrays.

Differential expression analysis was performed between samples reported in Table 1, using the pure Brain and Heart samples as a reference (see Methods). The correlation analysis between mixtures and reference samples quantifies the decrease in performance due to an increase in biological noise. Highest correlation coefficients highlight the robustness of the algorithms against biological noise.

The results of the analysis are reported in Tables 2, 3, 4, 5, 6 and 7. Tables 2 and 3 report the combination of affyILM with several summarization methods on old HG-U133Plus2 and new Hugene 1.0 ST arrays, respectively. For each combination, three differential expression analysis methods were used (Student t-test, Regularized t-test and Window t-test). More details can be found in **Methods**.

**Table 2:** Comparison of the differential expression analysis on HG-U133Plus2 arrays preprocessed with affyILM and popular summarization procedures

Summary	Data	**Diff. expr**.	m2 m8	m3 m7	m4 m6	m1 m6	m4 m9	m1 m4	m6 m9	m1 m5a	m5a m9	m1 m5b	m5b m9	m1 m5c	m5c m9
Average difference	Raw	Student	0.73	0.69	0.57	0.81	0.81	0.54	0.51	0.62	0.67	0.72	0.75	0.69	0.68
		Win. t	0.83	0.83	0.72	0.87	0.90	0.60	0.65	0.71	0.77	0.79	0.85	0.76	0.80
		Reg. t	0.75	0.81	0.67	0.84	0.89	0.56	0.61	0.72	0.72	0.78	0.82	0.76	0.77
	
	Scaled	Student	0.72	0.69	0.56	0.76	0.80	0.48	0.57	0.63	0.72	0.63	0.73	0.66	0.74
		Win. t	0.85	0.83	0.73	0.86	0.89	0.56	0.68	0.73	0.81	0.74	0.83	0.74	0.83
		Reg. t	0.81	0.79	0.67	0.83	0.87	0.49	0.63	0.68	0.76	0.69	0.79	0.69	0.78

Li-Wong MBEI	Raw	Student	0.71	0.69	0.57	0.78	0.77	0.53	0.45	0.61	0.68	0.69	0.74	0.69	0.67
		Win. t	0.81	0.82	0.72	0.84	0.86	0.58	0.62	0.69	0.77	0.77	0.83	0.75	0.79
		Reg. t	0.73	0.79	0.67	0.81	0.84	0.54	0.58	0.68	0.74	0.74	0.81	0.73	0.77
	
	Scaled	Student	0.68	0.65	0.52	0.68	0.73	0.43	0.54	0.57	0.65	0.54	0.67	0.60	0.67
		Win. t	0.81	0.79	0.70	0.80	0.83	0.51	0.66	0.66	0.76	0.67	0.79	0.67	0.77
		Reg. t	0.77	0.75	0.63	0.76	0.80	0.46	0.61	0.62	0.71	0.62	0.75	0.63	0.71

1-Step Tukey-Biweight	Raw	Student	0.78	0.74	0.64	0.85	0.84	0.61	0.51	0.66	0.71	0.77	0.79	0.76	0.71
		Win. t	0.86	0.86	0.77	0.90	0.92	0.65	0.68	0.73	0.80	0.83	0.89	0.80	0.83
		Reg. t	0.78	0.85	0.74	0.87	0.91	0.62	0.65	0.74	0.77	0.82	0.87	0.80	0.81
	
	Scaled	Student	0.76	0.73	0.61	0.78	0.83	0.54	0.63	0.65	0.74	0.66	0.76	0.71	0.77
		Win. t	0.87	0.86	0.78	0.89	0.92	0.61	0.74	0.74	0.83	0.77	0.86	0.77	0.85
		Reg. t	0.86	0.84	0.74	0.87	0.90	0.55	0.69	0.70	0.80	0.73	0.83	0.74	0.82

Median	Raw	Student	0.73	0.69	0.57	0.81	0.81	0.54	0.51	0.62	0.67	0.72	0.76	0.69	0.68
		Win. t	0.83	0.83	0.72	0.87	0.90	0.59	0.65	0.70	0.77	0.79	0.85	0.75	0.80
		Reg. t	0.76	0.81	0.68	0.85	0.89	0.55	0.61	0.70	0.74	0.77	0.83	0.75	0.78
	
	Scaled	Student	0.72	0.69	0.56	0.76	0.80	0.48	0.57	0.63	0.72	0.63	0.73	0.66	0.74
		Win. t	0.85	0.83	0.73	0.86	0.90	0.55	0.69	0.72	0.81	0.74	0.84	0.74	0.83
		Reg. t	0.82	0.80	0.68	0.83	0.87	0.50	0.64	0.68	0.77	0.70	0.80	0.70	0.79

Median-polish	Raw	Student	0.85	0.81	0.73	0.88	0.88	0.70	0.52	0.70	0.76	0.82	0.83	0.80	0.74
		Win. t	0.91	0.90	0.85	0.92	0.94	0.73	0.70	0.75	0.83	0.87	0.91	0.82	0.86
		Reg. t	0.90	0.89	0.86	0.93	0.94	0.74	0.70	0.75	0.85	0.88	0.90	0.83	0.87
	
	Scaled	Student	0.80	0.77	0.66	0.79	0.83	0.56	0.65	0.65	0.73	0.66	0.76	0.71	0.76
		Win. t	0.91	0.90	0.85	0.90	0.92	0.67	0.78	0.76	0.84	0.80	0.87	0.80	0.87
		Reg. t	0.93	0.91	0.86	0.91	0.94	0.65	0.80	0.76	0.86	0.79	0.89	0.81	0.90

We analyzed the HG-U133plus2 Tissue Mixture Study (Affymetrix) for differential expression, using Student t-test, Regularized t-test, and Window t-test. Summarization tests were performed on probe concentrations computed with AffyILM. Comparison between heart (mix 1) and brain (mix 9) is assumed to provide the reference list of p-values. For each combination of summarization/analysis steps (rows), Pearson's correlation coefficient has been computed on log10(p-values) between the reference analysis and several comparisons of mixtures (columns). Underlined characters highlight the top 5 correlation coefficients for each column. Raw/Scaled labels respectively refers to summarization tests performed with/without a scaling step between arrays.

**Table 3:** Comparison of the differential expression analysis on Hugene 1.0 ST arrays preprocessed with affyILM and popular summarization procedures

Summary	Data	**Diff. expr**.	m2 m8	m3 m7	m4 m6	m1 m6	m4 m9	m1 m4	m6 m9	m1 m5a	m5a m9	m1 m5b	m5b m9	m1 m5c	m5c m9
Average difference	Raw	Student	0.69	0.64	0.56	0.82	0.79	0.66	0.44	0.63	0.60	0.73	0.66	0.75	0.67
		Win. t	0.80	0.78	0.67	0.88	0.85	0.70	0.53	0.75	0.66	0.81	0.74	0.81	0.72
		Reg. t	0.66	0.66	0.54	0.82	0.79	0.63	0.48	0.73	0.59	0.77	0.67	0.75	0.58
	
	Scaled	Student	0.68	0.68	0.55	0.80	0.76	0.62	0.47	0.67	0.64	0.74	0.69	0.74	0.68
		Win. t	0.81	0.80	0.63	0.87	0.83	0.68	0.50	0.76	0.71	0.80	0.75	0.81	0.73
		Reg. t	0.70	0.70	0.51	0.80	0.76	0.59	0.41	0.66	0.59	0.71	0.64	0.73	0.65

Li-Wong MBEI	Raw	Student	0.74	0.68	0.62	0.84	0.79	0.69	0.45	0.63	0.64	0.75	0.70	0.76	0.72
		Win. t	0.82	0.79	0.71	0.86	0.83	0.70	0.53	0.71	0.68	0.80	0.77	0.79	0.74
		Reg. t	0.79	0.77	0.66	0.86	0.82	0.66	0.52	0.70	0.67	0.80	0.76	0.77	0.69
	
	Scaled	Student	0.71	0.70	0.58	0.77	0.74	0.61	0.46	0.63	0.63	0.72	0.68	0.71	0.68
		Win. t	0.81	0.81	0.65	0.83	0.80	0.66	0.50	0.72	0.70	0.78	0.75	0.77	0.72
		Reg. t	0.77	0.77	0.60	0.81	0.77	0.63	0.47	0.70	0.67	0.76	0.72	0.75	0.68

1-Step Tukey-Biweight	Raw	Student	0.77	0.71	0.66	0.87	0.83	0.72	0.48	0.67	0.66	0.78	0.73	0.80	0.73
		Win. t	0.84	0.82	0.73	0.91	0.88	0.74	0.57	0.76	0.70	0.83	0.79	0.83	0.76
		Reg. t	0.80	0.80	0.69	0.89	0.85	0.68	0.55	0.75	0.68	0.83	0.77	0.80	0.69
	
	Scaled	Student	0.76	0.75	0.62	0.84	0.80	0.67	0.53	0.70	0.69	0.78	0.73	0.78	0.73
		Win. t	0.84	0.84	0.68	0.88	0.85	0.72	0.55	0.78	0.74	0.83	0.78	0.83	0.77
		Reg. t	0.83	0.82	0.65	0.87	0.82	0.67	0.50	0.75	0.71	0.81	0.75	0.80	0.72

Median	Raw	Student	0.69	0.64	0.57	0.82	0.79	0.66	0.45	0.63	0.61	0.72	0.66	0.75	0.67
		Win. t	0.80	0.78	0.68	0.88	0.85	0.70	0.53	0.74	0.67	0.80	0.74	0.81	0.72
		Reg. t	0.77	0.76	0.63	0.86	0.82	0.65	0.50	0.73	0.64	0.79	0.72	0.78	0.66
	
	Scaled	Student	0.68	0.69	0.55	0.80	0.77	0.63	0.48	0.67	0.64	0.74	0.69	0.74	0.69
		Win. t	0.81	0.80	0.64	0.87	0.83	0.69	0.51	0.76	0.71	0.81	0.75	0.81	0.74
		Reg. t	0.78	0.77	0.59	0.85	0.80	0.63	0.45	0.72	0.67	0.77	0.72	0.77	0.69

Median-polish	Raw	Student	0.85	0.75	0.74	0.91	0.85	0.78	0.50	0.74	0.69	0.84	0.76	0.85	0.78
		Win. t	0.90	0.87	0.81	0.93	0.90	0.81	0.61	0.79	0.74	0.86	0.82	0.88	0.82
		Reg. t	0.91	0.87	0.83	0.95	0.90	0.83	0.62	0.80	0.75	0.88	0.84	0.90	0.82
	
	Scaled	Student	0.82	0.82	0.64	0.83	0.81	0.68	0.56	0.66	0.70	0.79	0.75	0.80	0.74
		Win. t	0.90	0.90	0.72	0.89	0.86	0.75	0.60	0.79	0.77	0.85	0.81	0.85	0.79
		Reg. t	0.91	0.91	0.76	0.92	0.89	0.77	0.62	0.82	0.80	0.87	0.84	0.87	0.81

We analyzed the Hugene 1.0 ST Tissue Mixture Study (Affymetrix) for differential expression, using Student t-test, Regularized t-test, and Window t-test. Summarization tests were performed on probe concentrations computed with AffyILM. Comparison between heart (mix 1) and brain (mix 9) is assumed to provide the reference list of p-values. For each combination of summarization/analysis steps (rows), Pearson's correlation coefficient has been computed on log10(p-values) between the reference analysis and several comparisons of mixtures (columns). Underlined characters highlights the top 5 correlation coefficients for each column. Raw/Scaled labels respectively refers to summarization tests performed with/without a scaling step between arrays.

**Table 4:** Comparison of the differential expression analysis between HG-U133Plus2 and Hugene 1.0 ST arrays preprocessed with affyILM and several summarization procedures

Summary	Data	**Diff. expr**.	m1 m9	m2 m8	m3 m7	m4 m6	m1 m6	m4 m9	m1 m4	m6 m9	m1 m5a	m5a m9	m1 m5b	m5b m9	m1 m5c	m5c m9
Average difference	Raw	Student	0.46	0.44	0.37	0.30	0.43	0.42	0.41	0.28	0.47	0.44	0.46	0.43	0.44	0.37
		Win. t	0.54	0.52	0.46	0.39	0.51	0.52	0.50	0.36	0.55	0.53	0.55	0.52	0.53	0.48
		Reg. t	0.47	0.43	0.37	0.35	0.46	0.46	0.48	0.29	0.50	0.44	0.48	0.43	0.48	0.42
	
	Scaled	Student	0.42	0.42	0.45	0.33	0.43	0.43	0.34	0.32	0.37	0.40	0.40	0.40	0.36	0.37
		Win. t	0.53	0.53	0.54	0.43	0.52	0.53	0.43	0.40	0.46	0.50	0.50	0.50	0.45	0.49
		Reg. t	0.46	0.46	0.49	0.39	0.45	0.48	0.39	0.34	0.38	0.42	0.41	0.42	0.39	0.43

Li-Wong MBEI	Raw	Student	0.47	0.40	0.38	0.33	0.45	0.46	0.41	0.32	0.47	0.48	0.47	0.46	0.43	0.38
		Win. t	0.52	0.49	0.44	0.42	0.50	0.53	0.49	0.38	0.53	0.56	0.53	0.54	0.50	0.50
		Reg. t	0.46	0.43	0.39	0.41	0.46	0.48	0.47	0.33	0.49	0.51	0.50	0.50	0.47	0.47
	
	Scaled	Student	0.38	0.39	0.43	0.35	0.40	0.43	0.32	0.34	0.33	0.41	0.36	0.42	0.32	0.39
		Win. t	0.49	0.49	0.52	0.44	0.49	0.53	0.40	0.43	0.42	0.51	0.46	0.52	0.43	0.52
		Reg. t	0.42	0.41	0.47	0.41	0.44	0.48	0.36	0.37	0.38	0.48	0.42	0.48	0.39	0.49

1-Step Tukey-Biweight	Raw	Student	0.52	0.50	0.42	0.36	0.49	0.49	0.50	0.36	0.54	0.54	0.53	0.52	0.52	0.43
		Win. t	0.57	0.56	0.48	0.44	0.55	0.57	0.57	0.42	0.61	0.61	0.60	0.59	0.58	0.54
		Reg. t	0.52	0.53	0.44	0.44	0.51	0.53	0.56	0.37	0.57	0.57	0.57	0.56	0.56	0.52
	
	Scaled	Student	0.45	0.45	0.48	0.37	0.45	0.47	0.40	0.39	0.41	0.45	0.43	0.46	0.41	0.42
		Win. t	0.56	0.56	0.58	0.47	0.55	0.58	0.49	0.47	0.50	0.56	0.53	0.57	0.50	0.56
		Reg. t	0.51	0.50	0.55	0.46	0.51	0.55	0.46	0.43	0.46	0.53	0.50	0.54	0.47	0.54

Median	Raw	Student	0.46	0.45	0.38	0.30	0.43	0.43	0.40	0.29	0.47	0.45	0.46	0.44	0.44	0.37
		Win. t	0.55	0.53	0.47	0.40	0.52	0.53	0.50	0.37	0.55	0.54	0.55	0.53	0.53	0.49
		Reg. t	0.49	0.49	0.42	0.39	0.48	0.49	0.48	0.31	0.52	0.49	0.52	0.48	0.49	0.46
	
	Scaled	Student	0.43	0.42	0.45	0.34	0.43	0.43	0.34	0.32	0.37	0.41	0.40	0.41	0.36	0.38
		Win. t	0.54	0.54	0.54	0.43	0.53	0.54	0.42	0.41	0.46	0.51	0.50	0.52	0.45	0.50
		Reg. t	0.48	0.48	0.51	0.42	0.48	0.50	0.38	0.35	0.41	0.47	0.45	0.48	0.42	0.48

Median-polish	Raw	Student	0.59	0.58	0.47	0.42	0.55	0.57	0.52	0.46	0.62	0.65	0.59	0.61	0.57	0.51
		Win. t	0.64	0.63	0.52	0.48	0.60	0.62	0.61	0.50	0.66	0.69	0.64	0.66	0.63	0.61
		Reg. t	0.64	0.64	0.52	0.51	0.61	0.62	0.62	0.51	0.67	0.68	0.64	0.67	0.64	0.61
	
	Scaled	Student	0.53	0.54	0.55	0.43	0.53	0.54	0.40	0.45	0.43	0.52	0.46	0.54	0.44	0.49
		Win. t	0.62	0.63	0.64	0.52	0.61	0.63	0.51	0.55	0.54	0.62	0.58	0.63	0.54	0.62
		Reg. t	0.62	0.62	0.64	0.55	0.62	0.64	0.53	0.56	0.55	0.64	0.59	0.65	0.55	0.64

We compared the results of the differential expression analysis of the Tissue Mixture Study between HG-U133plus2 and Hugene 1.0 ST arrays, using Student t-test, Regularized t-test and Window t-test. Preprocessing was performed with affyILM. For each combination of summarization/analysis steps (rows), and each comparison of mixtures (columns), Pearson's correlation coefficient has been computed on log10(p-values) between both types of arrays. Underlined characters highlight the top 5 correlation coefficients for each column. The best match table provided by Affymetrix has been used to map probesets between the two platforms. Raw/Scaled labels respectively refers to summarization tests performed with/without a scaling step between arrays.

**Table 5:** Comparison of the differential expression analysis on HG-U133Plus2 arrays preprocessed with popular methods

Method	Data	**Diff. expr**.	m2 m8	m3 m7	m4 m6	m1 m6	m4 m9	m1 m4	m6 m9	m1 m5a	m5a m9	m1 m5b	m5b m9	m1 m5c	m5c m9
MAS 5 1-Step Tukey-Biweight	Bg	Student	0.71	0.67	0.54	0.74	0.82	0.45	0.58	0.54	0.70	0.62	0.75	0.60	0.73
		Win. t	0.83	0.81	0.69	0.85	0.89	0.51	0.66	0.62	0.78	0.71	0.83	0.68	0.81
		Reg. t	0.83	0.79	0.68	0.85	0.88	0.45	0.61	0.57	0.77	0.69	0.83	0.65	0.78
	
	Bg	Student	0.80	0.79	0.69	0.83	0.86	0.62	0.69	0.78	0.82	0.78	0.82	0.79	0.83
	Norm	Win. t	0.89	0.88	0.79	0.90	0.92	0.66	0.73	0.84	0.88	0.84	0.88	0.84	0.88
		Reg. t	0.89	0.88	0.77	0.89	0.91	0.61	0.71	0.82	0.86	0.83	0.87	0.83	0.87
	
	PM	Student	0.72	0.70	0.56	0.77	0.85	0.52	0.59	0.57	0.72	0.68	0.80	0.62	0.73
		Win. t	0.82	0.82	0.72	0.87	0.93	0.59	0.69	0.66	0.80	0.77	0.88	0.68	0.80
		Reg. t	0.76	0.77	0.67	0.84	0.90	0.52	0.62	0.60	0.77	0.73	0.86	0.62	0.76
	
	PM	Student	0.84	0.82	0.73	0.86	0.89	0.66	0.75	0.77	0.84	0.79	0.85	0.82	0.86
	Norm	Win. t	0.91	0.90	0.83	0.92	0.94	0.69	0.79	0.82	0.88	0.85	0.91	0.86	0.91
		Reg. t	0.90	0.89	0.79	0.91	0.92	0.64	0.75	0.79	0.85	0.82	0.88	0.84	0.87

Plier	Raw	Student	0.73	0.71	0.56	0.75	0.82	0.57	0.56	0.64	0.70	0.71	0.76	0.67	0.71
		Win. t	0.82	0.80	0.70	0.83	0.87	0.60	0.63	0.67	0.74	0.76	0.82	0.70	0.77
		Reg. t	0.82	0.80	0.70	0.85	0.87	0.65	0.64	0.71	0.72	0.80	0.81	0.73	0.75

dChip Li-Wong MBEI	PM	Student	0.82	0.81	0.70	0.81	0.86	0.63	0.73	0.73	0.80	0.71	0.81	0.78	0.82
	Norm	Win. t	0.88	0.87	0.79	0.86	0.89	0.63	0.76	0.73	0.82	0.74	0.84	0.79	0.85
		Reg. t	0.88	0.86	0.73	0.85	0.88	0.58	0.73	0.70	0.78	0.70	0.81	0.77	0.81
	
	Norm	Student	0.80	0.79	0.72	0.80	0.84	0.59	0.71	0.74	0.80	0.73	0.81	0.74	0.80
		Win. t	0.86	0.85	0.79	0.84	0.87	0.61	0.73	0.76	0.82	0.76	0.84	0.77	0.83
		Reg. t	0.84	0.84	0.75	0.83	0.86	0.54	0.71	0.73	0.81	0.73	0.82	0.74	0.81

qFARMS	Norm	Student	0.81	0.80	0.72	0.81	0.85	0.65	0.71	0.73	0.79	0.73	0.81	0.77	0.81
		Win. t	0.85	0.84	0.73	0.81	0.84	0.54	0.64	0.69	0.77	0.70	0.79	0.71	0.78
		Reg. t	0.89	0.89	0.77	0.85	0.89	0.56	0.69	0.76	0.83	0.75	0.84	0.76	0.83

lFARMS	Norm	Student	0.81	0.79	0.70	0.79	0.84	0.62	0.64	0.72	0.73	0.72	0.78	0.76	0.80
		Win. t	0.85	0.84	0.73	0.81	0.84	0.54	0.60	0.68	0.72	0.70	0.76	0.70	0.77
		Reg. t	0.89	0.88	0.75	0.83	0.89	0.52	0.66	0.69	0.78	0.68	0.82	0.72	0.83

RMA Median-polish	Norm	Student	0.83	0.82	0.73	0.82	0.86	0.65	0.72	0.75	0.81	0.75	0.82	0.78	0.82
		Win. t	0.92	0.91	0.85	0.90	0.92	0.71	0.79	0.82	0.86	0.84	0.88	0.84	0.88
		Reg. t	0.93	0.93	0.87	0.92	0.93	0.73	0.79	0.84	0.88	0.86	0.90	0.86	0.89

GCRMA Median-polish	Raw	Student	0.85	0.84	0.76	0.83	0.89	0.57	0.72	0.49	0.78	0.61	0.84	0.69	0.83
		Win. t	0.90	0.88	0.84	0.90	0.92	0.62	0.75	0.57	0.80	0.68	0.86	0.73	0.85
		Reg. t	0.91	0.90	0.86	0.91	0.93	0.66	0.76	0.60	0.80	0.71	0.86	0.76	0.84
	
	Norm	Student	0.87	0.86	0.80	0.86	0.89	0.68	0.75	0.82	0.86	0.81	0.86	0.81	0.86
		Win. t	0.92	0.92	0.86	0.90	0.92	0.69	0.78	0.85	0.88	0.85	0.89	0.84	0.89
		Reg. t	0.94	0.94	0.89	0.92	0.95	0.71	0.79	0.87	0.91	0.87	0.92	0.86	0.91

We analyzed the HG-U133plus2 Tissue Mixture Study (Affymetrix) for differential expression, using Student t-test, Regularized t-test, and Window t-test. Several previously published preprocessing methods were compared. Comparison between heart (mix 1) and brain (mix 9) is assumed to provide the reference list of p-values. For each combination of preprocessing/analysis steps (rows), Pearson's correlation coefficient has been computed on log10(p-values) between the reference analysis and several comparisons of mixtures (columns). Underlined characters highlight the top 5 correlation coefficients for each column. Raw/Norm labels refer to raw and normalized data, PM stands for PM-only and Bg refers to background correction (using MM).

**Table 6:** Comparison of the differential expression analysis on Hugene 1.0 ST arrays preprocessed with several popular algorithms

Method	Data	**Diff. expr**.	m2 m8	m3 m7	m4 m6	m1 m6	m4 m9	m1 m4	m6 m9	m1 m5a	m5a m9	m1 m5b	m5b m9	m1 m5c	m5c m9
MAS 5 1-Step Tukey-Biweight	PM	Student	0.72	0.74	0.61	0.83	0.83	0.67	0.54	0.61	0.65	0.73	0.72	0.75	0.74
		Win. t	0.82	0.82	0.69	0.89	0.86	0.70	0.57	0.71	0.69	0.80	0.77	0.80	0.77
		Reg. t	0.79	0.81	0.64	0.87	0.83	0.65	0.56	0.67	0.69	0.78	0.77	0.76	0.72
	
	PM	Student	0.80	0.80	0.65	0.88	0.83	0.71	0.60	0.83	0.79	0.84	0.80	0.81	0.78
	Norm	Win. t	0.85	0.85	0.69	0.90	0.86	0.71	0.59	0.85	0.81	0.86	0.83	0.84	0.79
		Reg. t	0.86	0.85	0.65	0.90	0.84	0.67	0.57	0.83	0.80	0.85	0.82	0.82	0.75

Plier	Raw	Student	0.86	0.74	0.75	0.90	0.85	0.78	0.52	0.74	0.71	0.83	0.77	0.85	0.80
		Win. t	0.90	0.83	0.79	0.92	0.89	0.80	0.59	0.77	0.74	0.85	0.81	0.87	0.82
		Reg. t	0.91	0.84	0.81	0.94	0.89	0.83	0.60	0.79	0.74	0.87	0.82	0.89	0.82

dChip Li-Wong MBEI	PM	Student	0.80	0.74	0.65	0.86	0.84	0.71	0.49	0.63	0.71	0.77	0.77	0.79	0.77
	Norm	Win. t	0.85	0.80	0.71	0.88	0.86	0.72	0.54	0.69	0.73	0.81	0.81	0.81	0.78
		Reg. t	0.82	0.79	0.65	0.88	0.84	0.70	0.53	0.64	0.75	0.80	0.82	0.80	0.73

RMA Median-polish	Norm	Student	0.83	0.83	0.70	0.86	0.83	0.72	0.60	0.80	0.78	0.81	0.78	0.81	0.77
		Win. t	0.89	0.88	0.75	0.88	0.86	0.73	0.60	0.83	0.80	0.84	0.81	0.83	0.79
		Reg. t	0.93	0.92	0.80	0.93	0.90	0.77	0.63	0.87	0.84	0.89	0.85	0.88	0.84

GCRMA Median-polish	Raw	Student	0.84	0.86	0.76	0.82	0.88	0.53	0.64	0.41	0.69	0.75	0.76	0.69	0.81
		Win. t	0.87	0.89	0.79	0.86	0.87	0.56	0.60	0.49	0.68	0.80	0.75	0.73	0.79
		Reg. t	0.90	0.91	0.84	0.88	0.90	0.65	0.62	0.44	0.70	0.84	0.77	0.74	0.82
	
	Norm	Student	0.85	0.85	0.78	0.89	0.86	0.71	0.68	0.83	0.82	0.84	0.82	0.80	0.80
		Win. t	0.89	0.88	0.80	0.89	0.86	0.70	0.62	0.84	0.80	0.84	0.81	0.82	0.79
		Reg. t	0.94	0.94	0.86	0.95	0.92	0.75	0.66	0.89	0.86	0.90	0.86	0.88	0.84

We analyzed the Hugene 1.0 ST Tissue Mixture Study (Affymetrix) for differential expression, using Student t-test, Regularized t-test, and Window t-test. Several previously published preprocessing methods were tested. Comparison between heart (mix 1) and brain (mix 9) is assumed to provide the reference list of p-values. For each combination of preprocessing/analysis steps (rows), Pearson's correlation coefficient has been computed on log10(p-values) between the reference analysis and several comparisons of mixtures (columns). Underlined characters highlights the top 5 correlation coefficients for each column. Raw/Norm labels refer to raw and normalized data, PM stands for PM-only methods.

**Table 7:** Comparison of the differential expression analysis between HG-U133Plus2 and Hugene 1.0 ST arrays preprocessed with several popular algorithms

Method	Data	**Diff. expr**.	m1 m9	m2 m8	m3 m7	m4 m6	m1 m6	m4 m9	m1 m4	m6 m9	m1 m5a	m5a m9	m1 m5b	m5b m9	m1 m5c	m5c m9
MAS 5 1-Step Tukey-Biweight	PM	Student	0.51	0.49	0.46	0.36	0.48	0.52	0.49	0.40	0.52	0.55	0.54	0.56	0.49	0.45
		Win. t	0.60	0.57	0.54	0.46	0.56	0.62	0.59	0.48	0.61	0.63	0.63	0.64	0.58	0.58
		Reg. t	0.54	0.52	0.49	0.46	0.52	0.58	0.56	0.42	0.57	0.60	0.59	0.60	0.55	0.54
	
	PM	Student	0.48	0.49	0.49	0.42	0.50	0.50	0.44	0.40	0.44	0.46	0.48	0.47	0.45	0.49
	Norm	Win. t	0.57	0.57	0.59	0.53	0.59	0.61	0.55	0.51	0.55	0.57	0.58	0.58	0.55	0.60
		Reg. t	0.50	0.51	0.56	0.53	0.54	0.58	0.52	0.47	0.52	0.55	0.55	0.56	0.49	0.59

Plier	Raw	Student	0.51	0.59	0.48	0.41	0.52	0.49	0.40	0.43	0.57	0.59	0.56	0.56	0.46	0.49
		Win. t	0.58	0.64	0.54	0.49	0.59	0.58	0.52	0.51	0.62	0.64	0.62	0.62	0.56	0.60
		Reg. t	0.56	0.64	0.54	0.52	0.58	0.58	0.52	0.51	0.61	0.65	0.60	0.62	0.55	0.60

dChip Li-Wong MBEI	PM	Student	0.46	0.49	0.41	0.43	0.48	0.47	0.41	0.36	0.31	0.44	0.41	0.49	0.40	0.49
	Norm	Win. t	0.54	0.55	0.49	0.51	0.55	0.56	0.50	0.47	0.39	0.52	0.50	0.58	0.47	0.58
		Reg. t	0.48	0.51	0.45	0.51	0.53	0.52	0.46	0.41	0.30	0.49	0.45	0.55	0.41	0.55

RMA Median-polish	Norm	Student	0.54	0.55	0.55	0.46	0.52	0.54	0.44	0.46	0.45	0.52	0.48	0.52	0.49	0.52
		Win. t	0.64	0.64	0.64	0.56	0.62	0.65	0.56	0.58	0.57	0.64	0.59	0.63	0.59	0.64
		Reg. t	0.65	0.66	0.66	0.60	0.64	0.66	0.58	0.60	0.59	0.67	0.62	0.65	0.61	0.67

GCRMA Median-polish	Raw	Student	0.58	0.61	0.57	0.46	0.52	0.60	0.43	0.60	0.49	0.63	0.54	0.65	0.48	0.57
		Win. t	0.63	0.64	0.59	0.49	0.56	0.64	0.49	0.64	0.54	0.67	0.61	0.68	0.54	0.63
		Reg. t	0.63	0.65	0.60	0.52	0.56	0.65	0.52	0.65	0.52	0.68	0.62	0.69	0.52	0.64
	
	Norm	Student	0.50	0.49	0.48	0.46	0.49	0.51	0.47	0.51	0.47	0.50	0.48	0.51	0.47	0.49
		Win. t	0.60	0.59	0.59	0.55	0.57	0.61	0.54	0.60	0.56	0.61	0.58	0.60	0.56	0.61
		Reg. t	0.59	0.61	0.61	0.59	0.59	0.62	0.56	0.63	0.58	0.64	0.60	0.63	0.57	0.62

We compared the results of the differential expression analysis of the Tissue Mixture Study between HG-U133plus2 and Hugene 1.0 ST arrays, using Student t-test, Regularized t-test and Window t-test. Preprocessing was performed with several popular methods. For each combination of preprocessing/analysis steps (rows), and each comparison of mixtures (columns), Pearson's correlation coefficient has been computed on log10(p-values) between both types of arrays. Underlined characters highlight the top 5 correlation coefficients for each column. The best match table provided by Affymetrix has been used to map probesets between the two platforms. Raw/Norm labels refer to raw and normalized data, PM stands for PM-only methods.

The first three columns illustrate the correlations obtained when biological noise is added in equal amount in both samples. The following four columns illustrate the correlations computed when the analysis is performed between one sample (Brain or Heart) and the mixtures with 75% and 25% of each sample, respectively. The last six columns refer to the correlations measured on the comparisons between one sample (Brain or Heart), and 3 mixtures with 50% of each sample. The analysis shows that, whatever the differential expression analysis strategy, the best performances are obtained when the probe-level data is summarized using the medianpolish algorithm, followed by the 1-step Tukey-biweight algorithm. In addition, whatever the summarization method, the best results are obtained when the data is analyzed with variance-stabilizing methods (Regularized t-test and Window t-test), as compared to the classic Student t-test. As expected, the correlation decreases with increasing amounts of biological noise (column 1 to 3, column 4 to 8, 10, 12 to 6, and column 5 to 9, 11, 13 to 7). affyILM concentrations analyzed with or without the scaling step leads to similar results, and the observed correlation is higher using scaled data for some comparisons, lower for the others. The same conclusions can be formulated for both generation of arrays, as shown by comparing Table 2 and Table 3. The two types of arrays lead to similar performance results.

Table 4 summarizes the cross-platform correlation between the HG-U133Plus2 and Hugene 1.0 ST chips. For this purpose, we used the mapping table provided by Affymetrix (best match) [42] (See Methods). The correlations in Table 4 are lower than those reported in Tables 2 and 3, which means that the noise inherent to the platform discrepancies is higher than the noise observed between mixtures. Those discrepancies can be due to several causes. First, the definition of the probesets and the design of the probes does not follow the same strategy between 3' expression arrays and the recent Human Gene 1.0 array. Second, the mapping between the two arrays is not perfect, as a probeset from one array may be associated to several probesets from the other one. In the present study, we computed the scores using the best match mapping table provided by Affymetrix [42]. We only used unique mappings between the arrays, and discarded all the transcripts that are covered by only one of the two arrays. As a consequence, the cross-platform comparison is not perfect.

Using Table 4 to assess the performances of the individual methods leads to the same conclusions as from Tables 2 and 3: best results are obtained when affyILM is used in combination with the medianpolish summarization.

Tables 5, 6 and 7 report the correlations computed when the analysis is performed with current preprocessing methods, using the same 3 differential expression analysis methods. The highest correlations are observed when data is preprocessed using either RMA or GCRMA, both making use of the medianpolish summarization. The two methods provided by Affymetrix leads to lower correlations (MAS 5 and PLIER), and PLIER seems more appropriate than MAS 5 on the most recent array type (Tables 5 and 6). MAS 5 leads to better results on HG-U133Plus2, only when the mismatch probes are ignored (Table 5). Taken together, all those correlations tests suggest that the best methods are RMA, GCRMA and affyILM(medianpolish), whatever the array type.

As expected, a decrease in correlation is observed for increasing amounts of biological noise. However, the comparisons between mixes and Brain/Heart samples reveals an unexpected behavior: 50/50 mixes (mix5a, b, c) leads to higher correlation values when compared with Brain sample (mix9) than the correlation values obtained by comparison with Heart sample (mix1). One explanation for this effect could be that the 50/50 mixes are enriched in Heart sample, thus the difference between the mixes and Heart samples (mix1) may be lower than expected, and higher when compared to Brain samples (mix9). This effect can also be observed with 75% - 25% mixes (mix4 and mix 6) when compared to Brain and Heart samples (compare columns m1m6 with m4m9 or m1m4 with m6m9). As a consequence, we suspect that either the concentration of Heart samples was under-estimated, or the concentration of Brain samples was over-estimated, prior to the design of the mixes. Alternatively, this effect could be due to a difference in the quality of Heart and Brain samples, with a higher level of biological noise in the Brain sample than in the Heart sample (contamination, sample degradation, ...).

In the correlation study reported here, we tested affyILM in combination with the summarization steps used by other methods, to provide a fair comparison. The medianpolish summarization is associated to the best correlation coefficients, whichever preprocessing strategy is used. Giorgi *et al. *recently reported that the medianpolish procedure induce inter-array correlation, and introduced tRMA, where the use of rows and columns is inverted with regards to the medianpolish used in RMA and GCRMA (transposed medianpolish). This study also showed that the magnitude of the artifact associated with the medianpolish increases when the number of replicates is small, and is affected by an odd or even number of replicates [43]. In addition, in RMA and GCRMA, the medianpolish is performed on log-values (in accordance with the underlying model of RMA and GCRMA). We extended our correlation studies on differential expression results to compare the performances of the medianpolish and the transposed medianpolish, in combination with affyILM, and applied these procedures to the concentrations and to the log of the concentrations. The results of the analysis are supplied in additional files 1, 2 and 3 (respectively for HG-U133plus2, Hugene 1.0 ST and between array comparison). The transposed medianpolish and the medianpolish perform similarly, in agreement with Giorgi's statement that the reported artifact should not affect differential expression analysis. Our study also show that the medianpolish leads to higher correlation values when performed on log-values, in agreement with the model used by RMA and GCRMA.

### Performance evaluation on Latin-Square data

**Figure 1:**
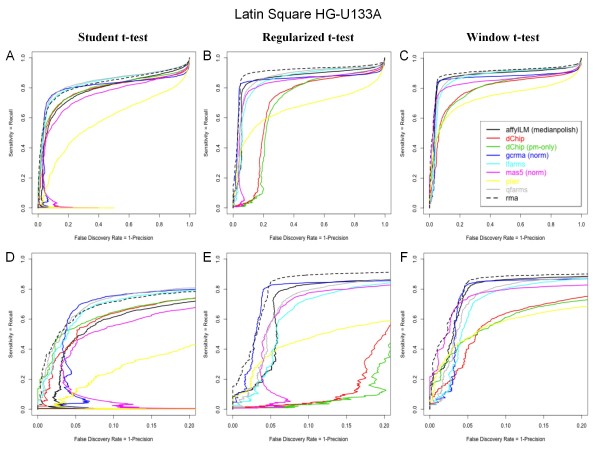
**Performance evaluation on Latin Square Data HG-U133A: Sensitivity VS False Discovery Rate**. The sensitivity (= TP/(TP+FN)) is compared to the false discovery rate (= 1-Precision = FP/(TP+FP)), using affyILM(medianpolish) and other popular preprocessing methods. A, B, and C respectively report the performance evaluation when the differential expression analysis is conducted with the student t-test, the window t-test, and the regularized t-test. D, E and F are zooms of A, B and C, in the lowest FDR region (up to 20%). Each curve is computed from the analysis of 91 available pairwise comparisons between 3 replicates of latin square samples.

**Figure 2:**
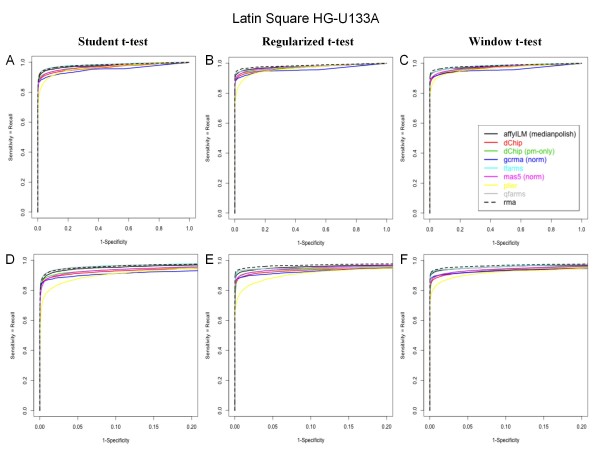
**Performance evaluation on Latin Square Data HG-U133A: ROC curves**. The sensitivity (= TP/(TP+FN)) is compared to 1-specificity (= FP/(TN+FP)), using affyILM(medianpolish) and other popular preprocessing methods. A, B, and C respectively report the performance evaluation when the differential expression analysis is conducted with the student t-test, the window t-test, and the regularized t-test. D, E and F are zooms of A, B and C, in the lowest 1-Specificity region (up to 20%). Each curve is computed from the analysis of 91 available pairwise comparisons between 3 replicates of latin square samples.

Correlation results on the differential expression analysis are not sufficient to discriminate those three methods. To best characterize the performance of the differential expression tests, we used the well-known latin-square HGU-133 spike-in dataset, which offers the opportunity to compare the most significant probesets with the precise knowledge of the true list of spiked RNA's. Each pairwise differential expression analysis has been performed, leading to 91 distinct comparisons (see Methods) [44]. The results from the 91 pairwise comparisons have been merged in a single list of p-values, and compared to the associated list of spiked genes to compute the sensitivity (= TP/(TP+FN)) and false discovery rate (FDR = 1-Precision = FP/(FP+TP)), depicted in Figure 1 using alternative ROC curves for each tested preprocessing and differential analysis methods. Those graphs are equivalent to Precison/Recall curves and compares the ability of the methods to find the truth with the price to pay for it, quantified by the proportion of errors in the top list, for increasing sizes of top-lists. The alternative ROC curves (Figure 1) can be used to best discriminate methods and can be interpreted similarly to traditional ROC curves (Figure 2, × axis = 1-specificity = FP/(TN+FP)), using the same Y axis, where the best performing methods are closer to the upper left corner of the graph (finding the truth without errors).

As demonstrated in several papers, variants of the Student t-test designed to stabilize the variance estimates outperforms the classic Student t-test, whichever preprocessing strategy is used [37,39,40,45-47]. The best performing individual analysis reported in our study is conducted with the window t-test. Comparing MAS5, RMA, GCRMA, PLIER and affyILM(medianpolish) reveals that the best performances for the window t-test and regularized t-test are obtained with RMA, affyILM(medianpolish) and GCRMA, as shown in Figure 1. Most of the spiked probesets are detected with less than 5%-10% of error in the top list with the window t-test or the reguralized t-test, and the remaining probesets are progressively detected. No method is able to detect all spiked RNA's. Using RMA, affyILM(medianpolish) and GCRMA in combination with the window t-test leads to a detection of more than 80% of the spiked genes with less than 5% of error in the top list. In conjunction with the window t-test, RMA and affyILM display a better top list than GCRMA, as the beginning of the curves is closer to the Y-axis. In the second part of those curves, the progressive detection of the remaining genes can be tracked by the error associated with the detection of 90% of the spiked genes: the corresponding FDR is close to 20%, 30% and 90% respectively for RMA, affyILM(medianpolish) and GCRMA. Using the classic Student t-test, the performances of affyILM(medianpolish) decreases and are lower than GCRMA, but still remains higher than PLIER and MAS 5.0.

Figure 2 illustrates the performances of the analysis with regard to the specificity (traditional ROC curves). All the methods quickly reaches a high level of sensitivity. However, affyILM(medianpolish), RMA and FARMS are able to reach a higher sensitivity level, followed by dChip, as compared to GCRMA. The most interesting part of those curve, featuring low 1-specificity values (high specificity), illustrates that the best performances are obtained with RMA, affyILM and FARMS (Figure 2D-F).

The AffyComp assessment of preprocessing methods allow to compare the performances of preprocessing methods by monitoring several descriptive statistics. Although affyILM misses an appropriate background correction algorithm, we submitted affyILM(medianpolish) to the AffyComp III assessment. The results of the assessment will serve as a basis for the evaluation of affyILM future developments. The aim of this paper was to assess the performances of affyILM with regards to differential expression. However, we provide the reports of Affycomp using affyILM(medianpolish) in the additional files 4 and 5, respectively for the Latin Squares HG-U95a and HG-U133. Additional files 6 and 7 summarize the AffyComp III scores of affyILM and selected methods. The scores obtained reveal that methods can be splitted in two categories, and best methods are RMA, GCRMA, FARMS and affyILM, in accordance with our performance evaluation.

## Conclusions

The aim of this paper was to perform a thorough performance analysis of affyILM, a Bioconductor package designed to preprocess Affymetrix GeneChip expression data. This model relies on the thermodynamics of hybridization and avoids complex statistical transformation as normalization steps used by other methods. To avoid biases due to the variability of the amount of hybridized material, the concentrations are scaled with an array-specific factor (selected to get a slope of 1 between pairwise array comparisons). To avoid biases due to the need for platform-specific background estimation algorithms, the background correction step has been ignored in our study. The study reported here adresses two main goals: evaluating the performances of affyILM with respect to differential expression analysis, and selecting the best summarizing strategy to avoid outliers-associated bias.

Our correlation study on mixtures between two biological tissue samples first highlights that the medianpolish summarization leads to the best results in conjunction with the extended Langmuir Isotherm, followed by 1-step Tukey-Biweight algorithm and MBEI, as seen from the data shown in Tables 2, 3 and 4. The comparison with other methods reveals that the correlations observed in our study are similar to the best performing methods. The performances are similar for HG-U133 plus 2 and the recent Hugene 1.0 ST array type. The performance evaluation has been completed by an analysis of the HG-U133 Latin Square experiment, allowing to compare the most significant genes with the true knowlegde of spiked RNAs. The package affyILM used in combination with variants of the Student t-test that stabilizes the variance, provides a better top-list than GCRMA, and is close to RMA for this dataset. The three best methods relies on the medianpolish summarization, highlighting the importance of the summarization step. Using the traditional t-test, performances of affyILM are lower, in agreement with our expectations, due to the absence of a background-correction step.

According to the statistical tests performed in this paper the accuracy of affyILM is similar to the best performing preprocessing algorithms (RMA, GCRMA...). The advantages of affyILM is that it is entirely based on physical principles and does not make use of excessive parameters fitting. It runs equally well on a single experiment and it does not make use of heavy normalization, apart from global rescaling of the concentration levels. In addition, it provides to each measured expression level an error estimate [28], which is useful to discriminate between the robust determined expression levels, from those with high error rates. The experience with other type of arrays [48] suggests that the performance of affyILM could be further increased with a better parametrization of the hybridization free energies, which are used by affyILM to compute the concentrations from the Langmuir isotherm. So far the free energy values used are taken from Sugimoto *et al. *data [49], obtained from experiments of hybridization between RNA and DNA strands in solution.

In the future, our efforts will be shared on two objectives. First, we will try to further improve the strategy by including a background-correction step. In our current implementation of affyILM, this step is not performed, causing a variable over-estimation of the concentration. Second, we will try to simplify the analysis by testing an appropriate weighted multivariate analysis strategy from probe concentrations, instead of summarizing it. Transcript concentrations are estimated from each probe, to get rid of a dependance between the intensity and the sequence-specific hybridization free energies. As estimated target concentrations are analysed in place of probe intensities, all probes targetting specificaly the same transcript should thus provide the same information and share the same biological variability. Multivariate analysis procedures are typically used to analyse such data. This strategy should be more powerful, as a multivariate analysis uses more values in the test than an univariate analysis of summarized values. This strategy was previously used by Barrera *et al. *on intensity values and proved to be efficient [50]. However, the univariate analysis reported here on summarized values shows that affyILM performs best in combination with the medianpolish, which highlights the impact of outlying probes during the summarization step. Outlying probes reveal the presence of unexpected behavior (cross-hybridization, errors in probeset definition or probe sequence...). To avoid biases during multivariate analysis in the presence of outliers, we will focus on the definition of appropriate weighting factors for each probe.

## Methods

### Datasets

We first selected the tissue mixture study dataset provided by Affymetrix in order to characterize the correlation results of the differential expression analysis between brain and heart samples, and several mixtures of the two samples, as described in Table 1. Each tissue mixture was hybridized on two distinct generation of expression arrays, namely the 3' HG-U133Plus2 expression array and the Human Gene 1.0 ST v1 array. Each sample/mixture was hybridized on 3 arrays (triplicates) [41]. In order to characterize the performances of several preprocessing algorithms (in combination with several differential expression analysis methods), we followed two distinct strategies. In the first strategy, we computed Pearson's correlation coefficient between the significance of the differential expression analysis performed on the optimal comparison (Brain VS Heart = Mix1 vs Mix9) and on several mixtures comparisons (Mix.x vs Mix.y), thus comparing the results with increasing biological noise. The differential expression analysis, as well as the preprocessing strategy, are described in the Procedures section hereunder. In the second strategy, the correlations were computed between the two generations of arrays, for each available pairwise mixture comparison. This second strategy was used to compare the performances of each analysis strategy/preprocessing method for their ability to extract common information from both array type.

To best characterize the performances of the selected preprocessing/analysis strategies, we selected the latin-square HG-U133A spike-in dataset. The design of the latin-square experiment relies on the definition of 14 sets of 3 probesets, leading to 42 probesets spiked with known concentrations of RNA. Using those RNAs, 14 triplicated hybridizations were performed with increasing concentrations (0, 0.25, 0.5, 1, 2, 4, 8, 16, 32, 64, 128, 256, 512 and 1024 pM). Each set of 3 probesets is spiked with a specific concentration. The latin-square experiment leads to 91 possible pairwise comparisons between triplicate experiments [44]. The Bioconductor package AffyComp [36] provides a list of probe sets which potentially cross-hybridize with the spiked transcripts of the Latin square HGU-133A experiment. To avoid cross-hybridization biases, these probesets have been discarded from our analysis, in agreement with the procedure implemented in the AffyComp package. Some of these probesets are expected to hybridize to the spiked sequences, implying that we must consider them as true positives if analyzed, but this would be a violation of the latin-square design (14 groups of 3 probesets spiked in 14 concentrations). As an example, the spiked clone sequence of probeset AFFX-ThrX-3_at aligns with probes defining probesets AFFX-ThrX-5_at, AFFX-ThrX-M_at, AFFX-r2-Bs-thr-3_s_at, AFFX-r2-Bs-thr-5_s_at and AFFX-r2-Bs-thr-M_s_at. The Affymetrix description document of the Latin Square HG-U133a also refers to 3 probesets known to cross-hybridize (included in the probesets listed by AffyComp).

### Procedures

The raw data provided by Affymetrix was preprocessed using the R programming environment and some Bioconductor packages [51,52]. The estimation of probe-level concentrations from the Langmuir isotherm was performed using affyILM. The summarization of probe-level concentrations was performed using internal functions of the affy package [53], by first creating an affybatch with the probe-level concentrations. GCRMA was performed using the gcrma package. The GCRMA methodology makes use of MM probes to estimate the background distribution for each array [11]. In the Human Gene 1.0 st data (PM-only arrays), the background distribution was computed using the set of anti-genomic probes designed by Affymetrix. MAS 5.0, RMA and dChip were performed with the expresso function of the affy package. PLIER and FARMS were respectively performed with the R/Bioconductor packages named plier and farms.

Differential expression analysis was performed using PEGASE, an R package written by Berger et al. in order to use several methods and to compare the results [54]. Probesets containing only NA values in at least one of the 2 subsets of each pairwise comparison were removed. Selected methods are the classic Student t-test, the Regularized t-test [40], and the Window t-test [38].

For each available pairwise comparison in the Tissue Mixture study, the lists of p-values generated by PEGASE were used to compute a correlation score using two strategies.

In the first strategy, we first defined the Mix1 vs Mix9 (Brain VS Heart) comparison as the reference list of p-values, for each type of array and for each analysis strategy. Pearson's correlation coefficient was then computed between the log10 of the reference p-values and the log10 of the p-values associated to any other pairwise comparison between mixtures. This procedure was repeated for each tested combination of preprocessing and differential expression analysis methodologies.

The aim of the second strategy is to compare results from HG-U133Plus2 and Human Gene 1.0 ST v1 arrays. First, we retrieved the mapping table between the probesets designed in the two generations of arrays, available in the support documentation of the supplier. We selected the best-match mapping table, and used it to subset the list of p-values obtained from both types of array [42]. Pearson's correlation coefficient was then computed on the mapped subsets, for a given pairwise comparison between mixtures, between the log10 of the lists of p-values computed from both generation of arrays. The procedure was repeated for each tested combination of preprocessing and differential expression analysis methods that are compatible with the two types of arrays, and for each available pairwise comparison.

The performance evaluation of the Latin-square data was done using the PEGASE R package [54]. As 14 samples define the latin-square experiment, 91 pairwise comparisons can be performed between triplicates (see **Datasets **section above). The differential expression analysis was performed with PEGASE (Student t-test, Regularized t-test and Window t-test) and 91 lists of p-values were generated for each method. To quantify the performances of the analysis in one step, the 91 lists of p-values were concatenated in a single list, for each combination of preprocessing/analysis methods. In addition, known concentrations of spiked RNA's were used to compute 91 lists of fold-change values (FC). The other probesets defined on the array are not expected to vary between samples and were associated to a fold-change value equal to 1. The 91 lists of expected fold-change were then converted into 91 binary lists (True if FC < 1 or FC > 1; False if FC = 1), and concatenated in a single binary list. For each combination of preprocessing and differential expression analysis methodologies, the final binary list of spiked probesets and the total list of p-values were used with PEGASE to compute the sensitivity (= recall = TP/(TP+FN)), the false discovery rate (FDR = 1-precision = FP/(FP+TP)), and 1-specificity (= FP/(TN+FP)) for increasing thresholds.

## Authors' contributions

FB scripted the procedures and ran the analysis of the data. Both authors contributed to the analysis, interpretation and redaction of the paper. All authors read and approved the final manuscript.

## Supplementary Material

Additional file 1 **HG-U133Plus2-medianpolish-comp**. HG-U133Plus2-medianpolish-comp.xls summarizes the performances of affyILM with the transposed medianpolish on the HG-U133 Plus 2 arrays (Tissue mixture correlation study). Click here for file

Additional file 2 **Hugene10st-medianpolish-comp**. Hugene10st-medianpolish-comp.xls summarizes the performances of affyILM with the transposed medianpolish on the Hugene 1.0 ST arrays (Tissue mixture correlation study). Click here for file

Additional file 3 **HG-U133Plus2-VS-Hugene10st-medianpolish-comp**. HG-U133Plus2-VS-Hugene10st-medianpolish-comp.xls summarizes the performances of affyILM with the transposed medianpolish using cross-platform comparisons between HG-U133Plus 2 and Hugene 1.0 ST arrays. (Tissue mixture correlation study). Click here for file

Additional file 4 **Affycomp-LS95-report**. affycomp-LS95-report.pdf provides the report of the affycomp III evaluation on the latin square HG-U95 experiment. Click here for file

Additional file 5 **Affycomp-LS133-report**. affycomp-LS133-report.pdf provides the report of the affycomp III evaluation on the latin square HG-U133 experiment. Click here for file

Additional file 6 **Affycomp-LS95-scores**. affycomp-LS95-scores.xls reports the scores assessed by affycomp III evaluation of selected methods, on the latin square HG-U95 experiment. Click here for file

Additional file 7 **Affycomp-LS133-scores**. affycomp-LS133-scores.xls reports the scores assessed by affycomp III evaluation of selected methods, on the latin square HG-U133 experiment. Click here for file
