# Supplementary material for: From hybridization theory to microarray data analysis: performance evaluation
Source: BMC Bioinformatics. 2011 Dec 2;12:464. doi: 10.1186/1471-2105-12-464 (PMC3267830; doi:10.1186/1471-2105-12-464)
Supplement: Additional file 4 — Affycomp-LS95-report. affycomp-LS95-report.pdf provides the report of the affycomp III evaluation on the latin square HG-U95 experiment. [file 1471-2105-12-464-S4.PDF]

Bioconductor Expression Assessment Tool for Affymetrix Oligonucleotide Arrays (affycomp)

September 26, 2011

Contents

In this report only assessment using the **HGU95** spike-In experiment are presetned. Figures 2,3, and 4b are therefore ommited.

```
fullname: Fabrice Berger
method: affyILMnoBGmedpolish
nickname: ILMpmMDP
competition: NO

Overall signal to ratio assessment:

             slope       R2   medianSD Null FC IQR Null FC 99.9% Rank
ILMpmMDP 0.4948024 0.666954 0.04189417  0.06735429     0.3130504    2

Expected Rank (out of 12626) for genes with fold change of 2
when all other genes are not differentially expressed
stratified by nominal concentration.

         ILMpmMDP
0.25:0        266
0.5:0.25     4212
1:0.5         154
2:1            47
4:2             6
8:4             3
16:8            3
32:16           2
64:32           2
128:64          2
```

256:128	2
512:256	2
1024:512	3

Table 1 for Spike-in Data:

	ILMpmMDP
Signal detect slope	0.5036107
Signal detect R2	0.6760342
AUC (FP<10)	0.5456701
AUC (FP<15)	0.5971671
AUC (FP<25)	0.6631674
AUC (FP<100)	0.8058646
AFP, call if fc>2	1.6851852
ATP, call if fc>2	10.5041152
IQR	0.1585088
Obs-intended-fc slope	0.4923114
Obs-(low)int-fc slope	0.1419499
FC=2, AUC (FP<10)	0.3059349
FC=2, AUC (FP<15)	0.3588898
FC=2, AUC (FP<25)	0.4214154
FC=2, AUC (FP<100)	0.5758929
FC=2, AFP, call if fc>2	1.2500000
FC=2, ATP, call if fc>2	2.3214286

Table 2 for Spike-in Data:

	ILMpmMDP
null log-fc IQR	0.06735429
null log-fc 99%	0.18926707
null log-fc 99.9%	0.31305037
low AUC	0.34707611
med AUC	0.84220297
high AUC	0.85968441
weighted avg AUC	0.47103264
25% SD	0.03252085
Median SD	0.04189417
75% SD	0.05438442
99% SD	0.16791969
low.slope	0.12769464
med.slope	0.51354825
high.slope	0.61974645
low.R2	0.01807385

```
med.R2          0.24657705
high.R2         0.24789247
0.25:0          0.13139422
0.5:0.25        0.02126221
1:0.5           0.15336013
2:1             0.19990640
4:2             0.31619919
8:4             0.41731711
16:8            0.49693114
32:16           0.63193553
64:32           0.70777181
128:64          0.81695683
256:128         0.61660153
512:256         0.62292842
1024:512        0.42248048
```

Figure 1

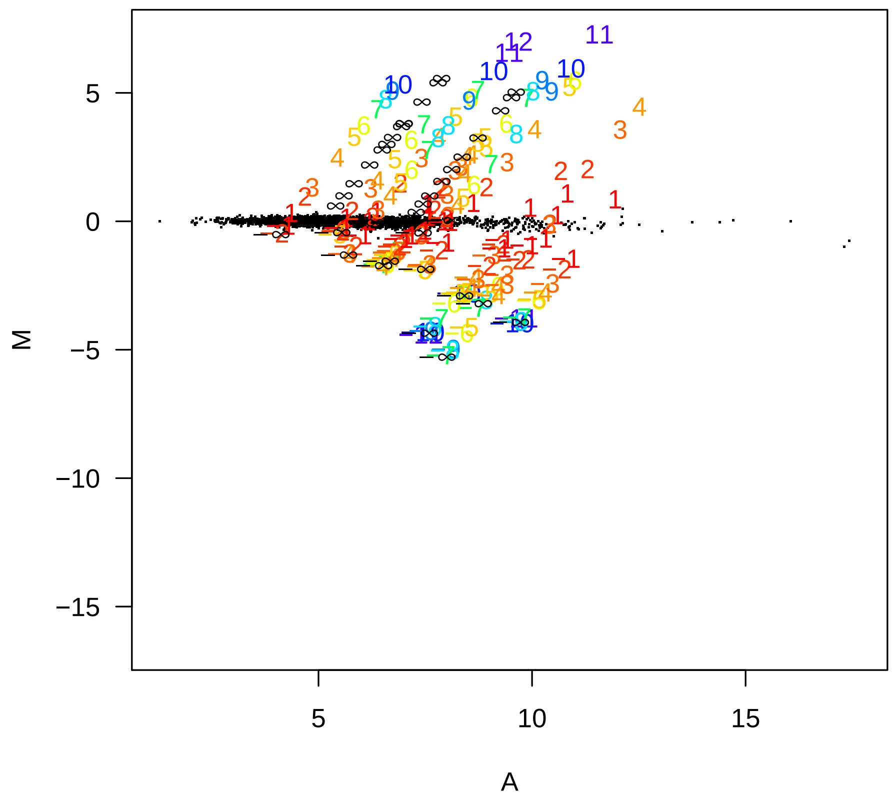

Figure 1: The MA plot shows log fold change as a function of mean log expression level. A set of 14 arrays representing a single experiment from the Affymetrix spike-in data are used for this plot. A total of 13 sets of fold changes are generated by comparing the first array in the set to each of the others. Spiked-in genes are symbolized by numbers representing the nominal $\log_2$ fold change for the gene. Non-differentially expressed genes with observed fold changes larger than 2 are plotted in red. All other probesets are represented with black dots.

Figure 1b

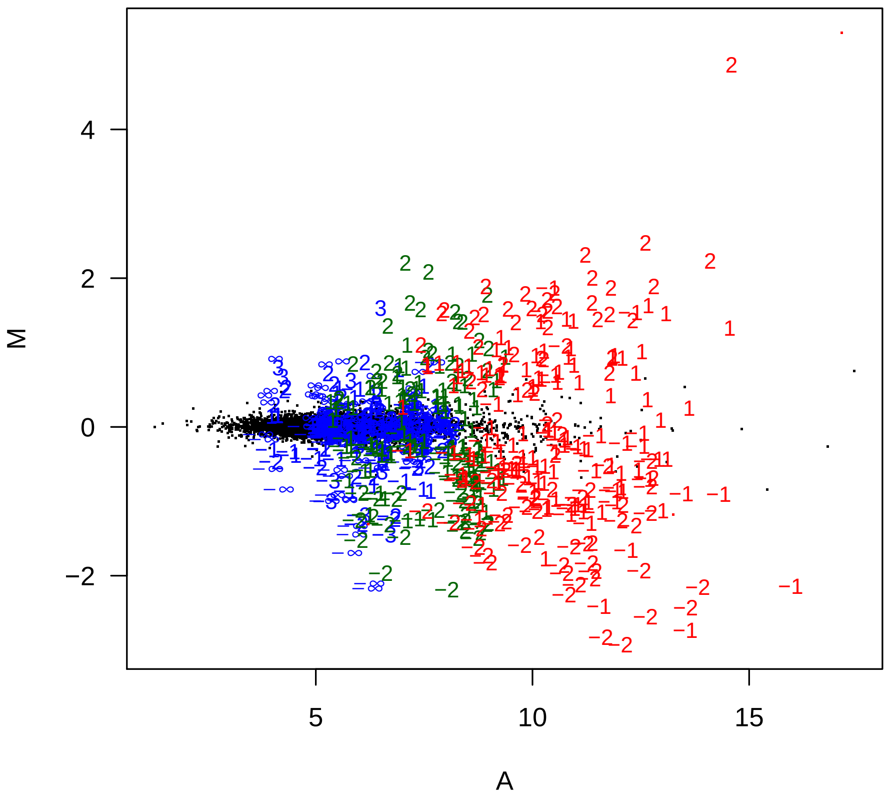

Figure 1b: The MA plot shows log fold change as a function of mean log expression level. A set of 28 arrays representing a single experiment from the Affymetrix spike-in data are used for this plot. Fold changes are generated for all possible comparisons of the the first 14 arrays and the second 14 arrays. Spiked-in genes are symbolized by numbers representing the nominal $\log_2$ fold change for the gene. Of the genes that are spiked to be differentially expressed, only genes with small nominal fold changes are shown. The colors represent four different groups: nominal concentration of genes being compared less than or equal to 2 picoMolar (blue), between 4 and 32 picoMolar (green), greater than or equal to 64 picoMolar (blue). Non-differentially expressed genes with observed fold changes larger than 2 are plotted in red. All other probesets are represented with black dots.

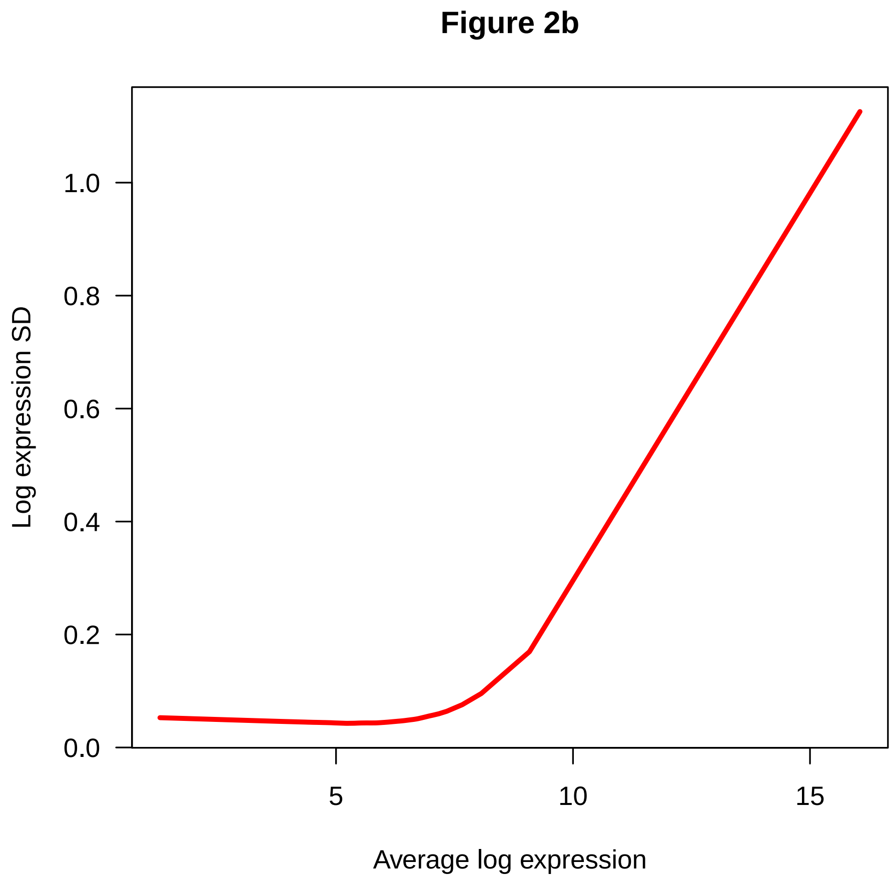

Figure 2b: For each non-spiked-in gene in the 28 arrays used in Figure 1b, we calculate the mean log expression and the observed standard deviation across the 28 replicates. The resulting scatterplot is smoothed to generate a single curve representing mean standard deviation as a function of mean log expression.

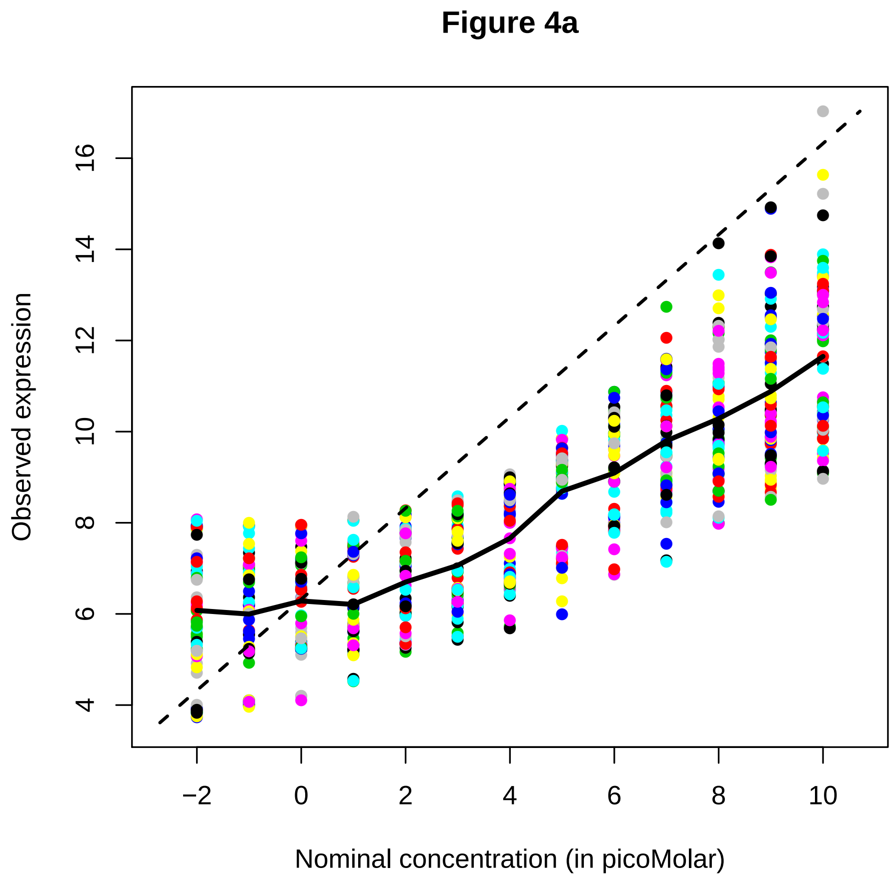

Figure 4a) Average observed log_2 intensity plotted against nominal log_2 concentration for each spiked-in gene for all arrays in Affymetrix spike-In experiment. The dashed line has the ideal slope of 1.

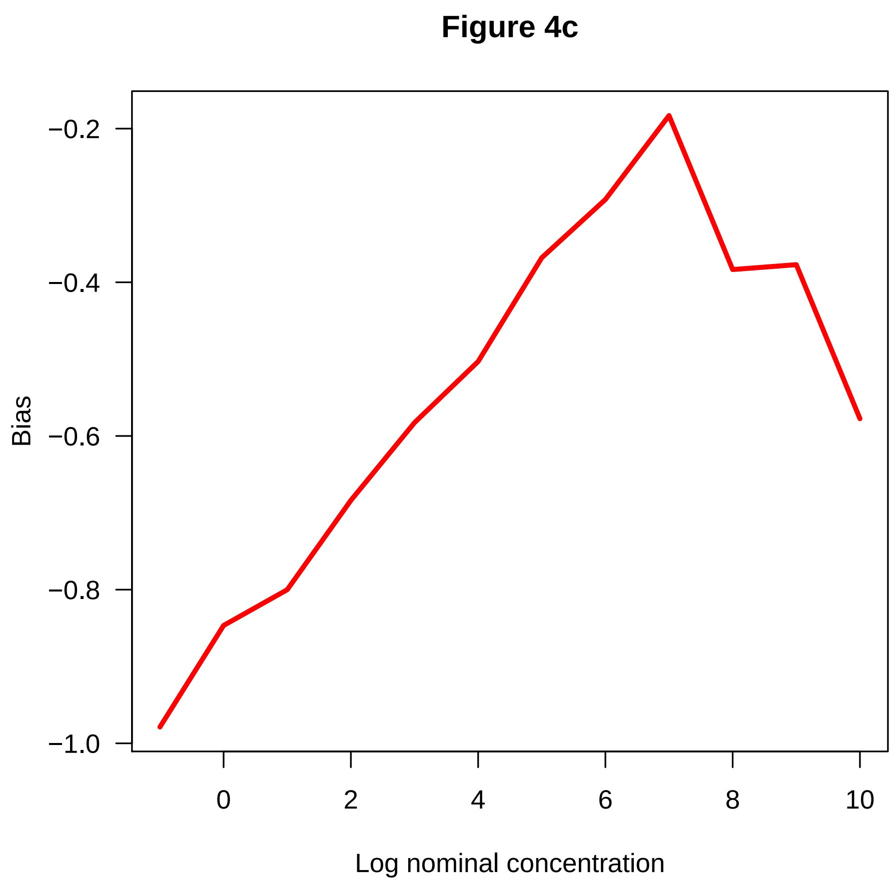

Figure 4c) Using the 28 arrays of Figure 1b, we compute local slopes. As the slopes shown in Figure 4a), the local slopes represent the expected observed log fold-change for probesets with true fold-change of 2 but they are presented as a function of the total nominal probeset concentration in the two samples being compared. In theory the local slopes should be one so we show the bias (difference between the observed local slope and one).

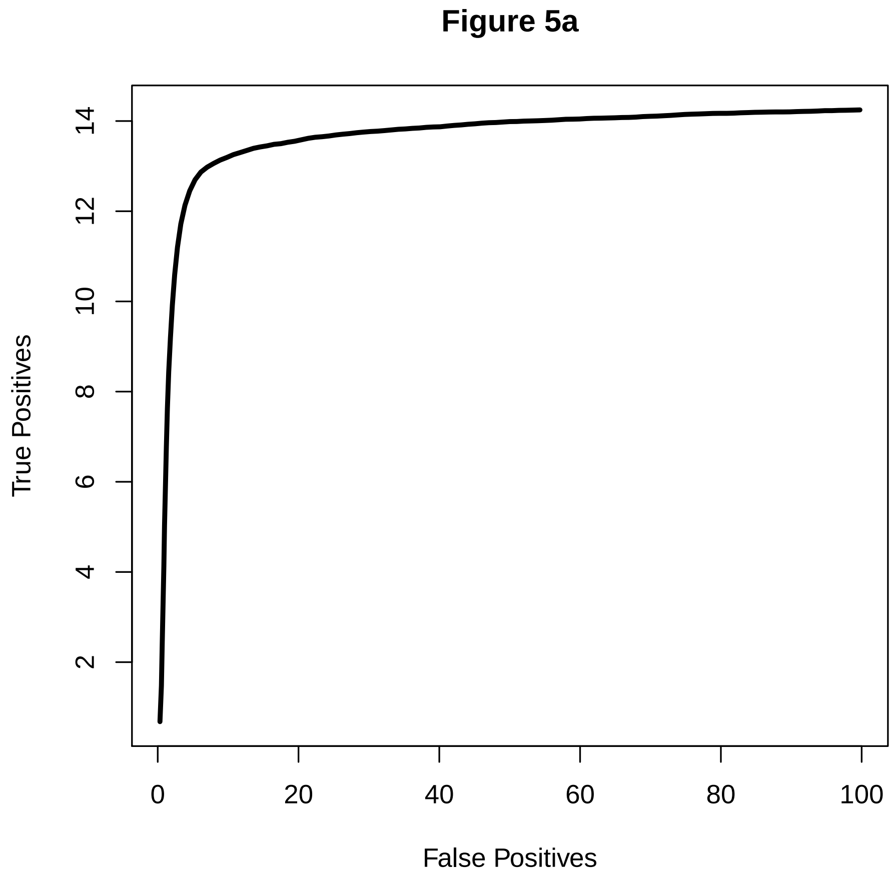

Figure 5a) A typical identification rule for differential expression filters genes with fold change exceeding a given threshold. This figure shows average ROC curves which offer a graphical representation of both specificity and sensitivity for such a detection rule. Average ROC curves based on comparisons with nominal fold changes ranging from 2 to 4096.

Figure 5b

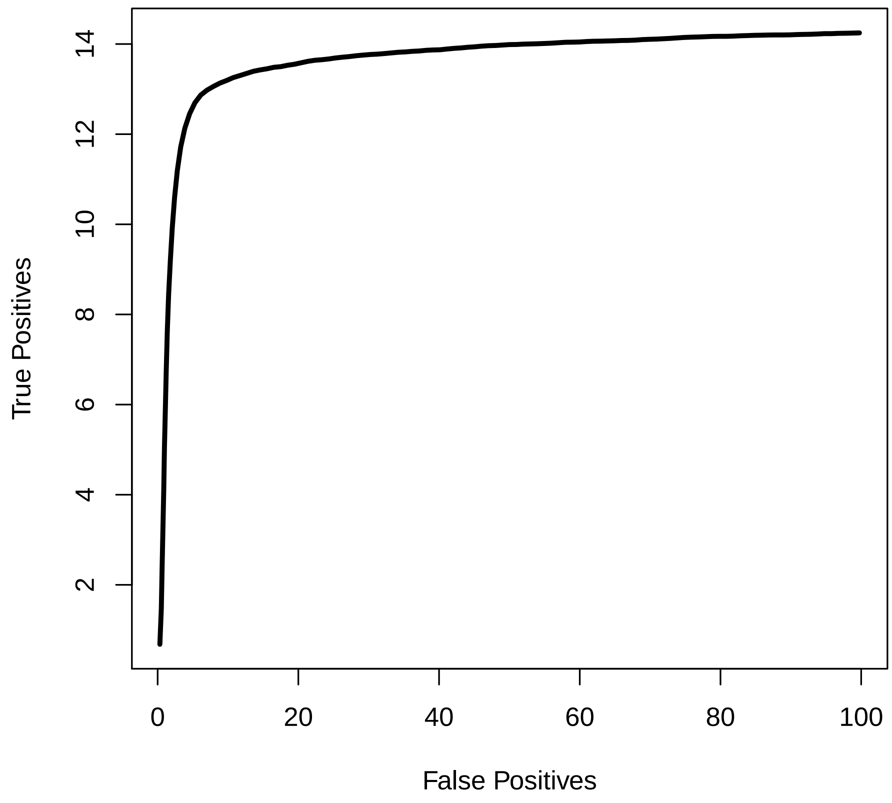

Figure 5b) As 5a) but with nominal fold changes equal to 2.

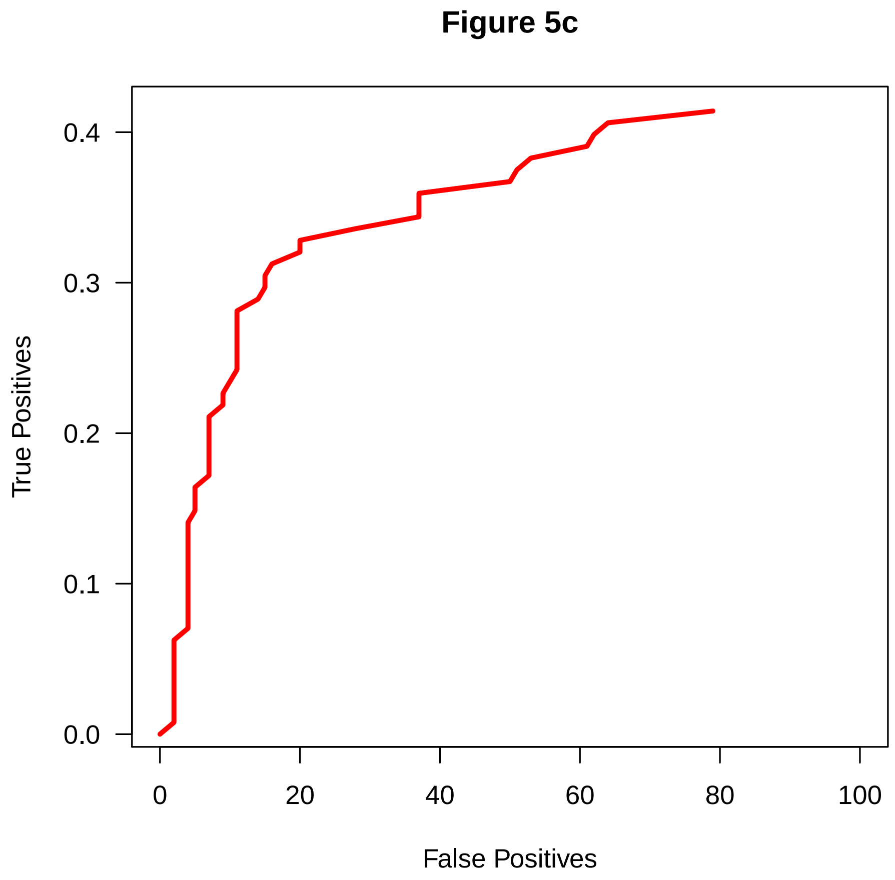

Figure 5c) As 5a) but for comparisons with both nominal concentrations at most 4 picoMolar and nominal fold changes at most 2.

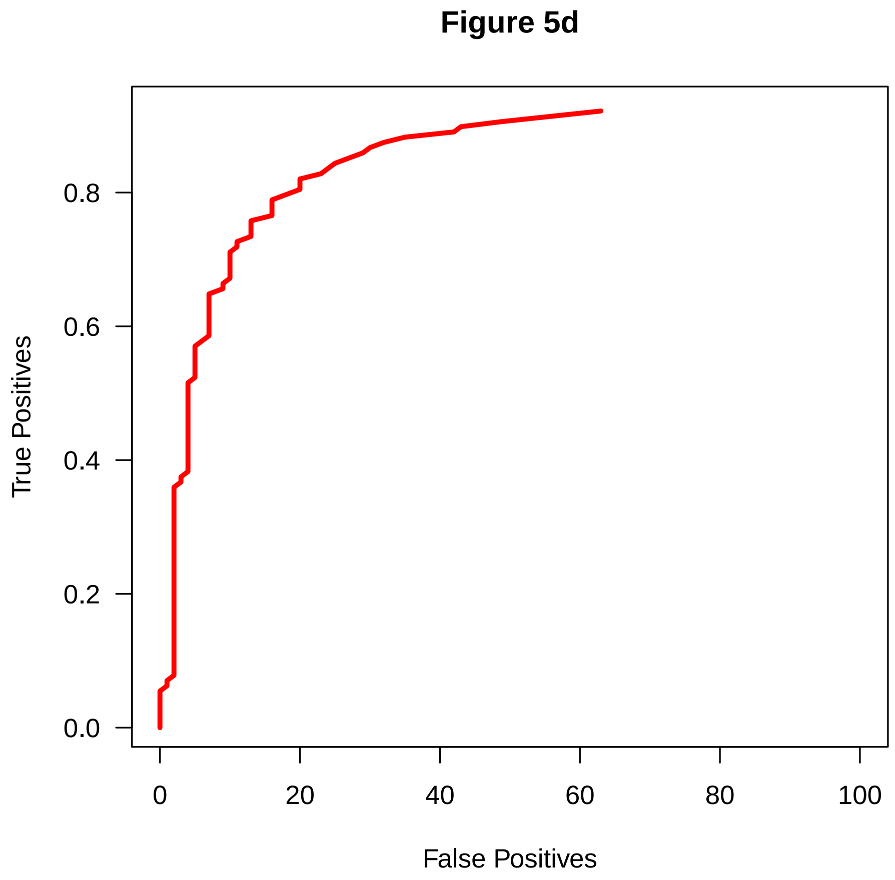

Figure 5d) As 5a) but for comparisons with both nominal concentrations between 4 and 64 picoMolar and nominal fold changes at most 2.

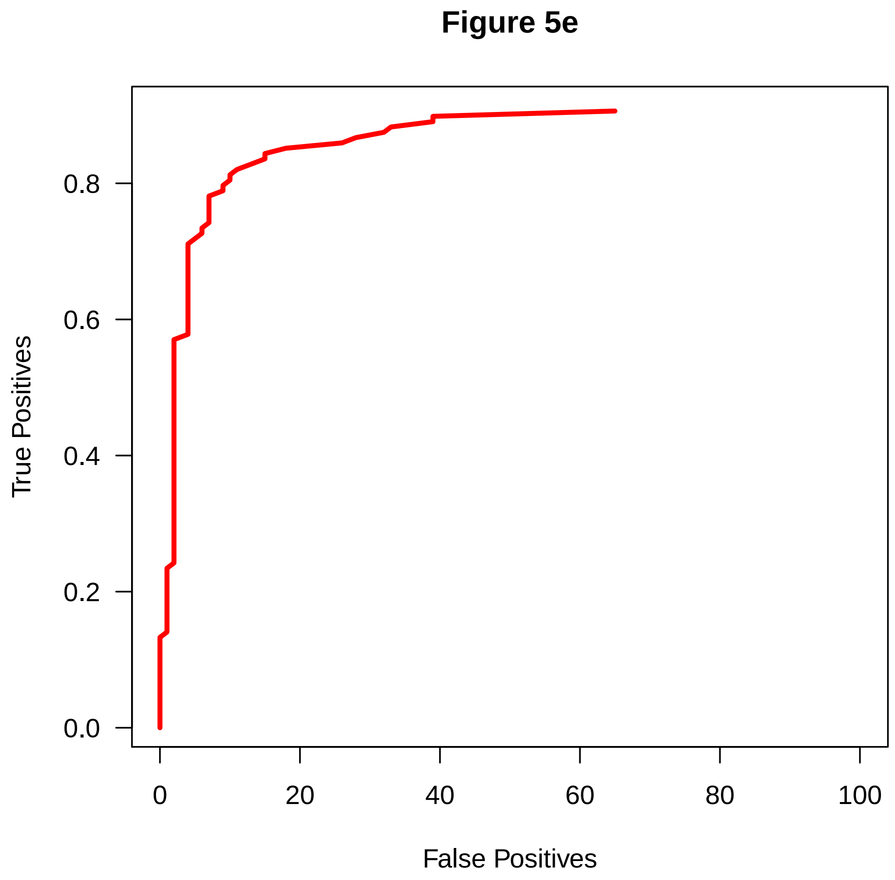

Figure 5e) As 5a) but for comparisons with both nominal concentrations at least 64 and with nominal fold changes at most 2.

Figure 6a

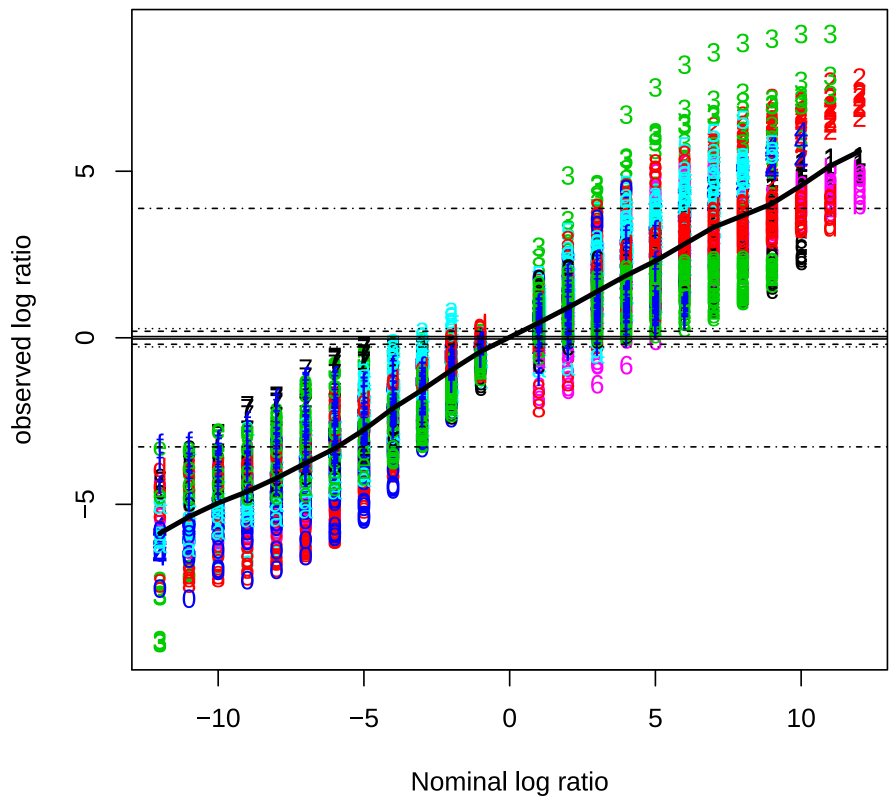

Figure 6a) Observed log fold changes plotted against nominal log fold changes. The dashed lines represent highest, 25th highest, 100th highest, 25th percentile, 75th percentile, smallest 100th, smallest 25th, and smallest log fold change for the genes that were not differentially expressed.

Figure 6b

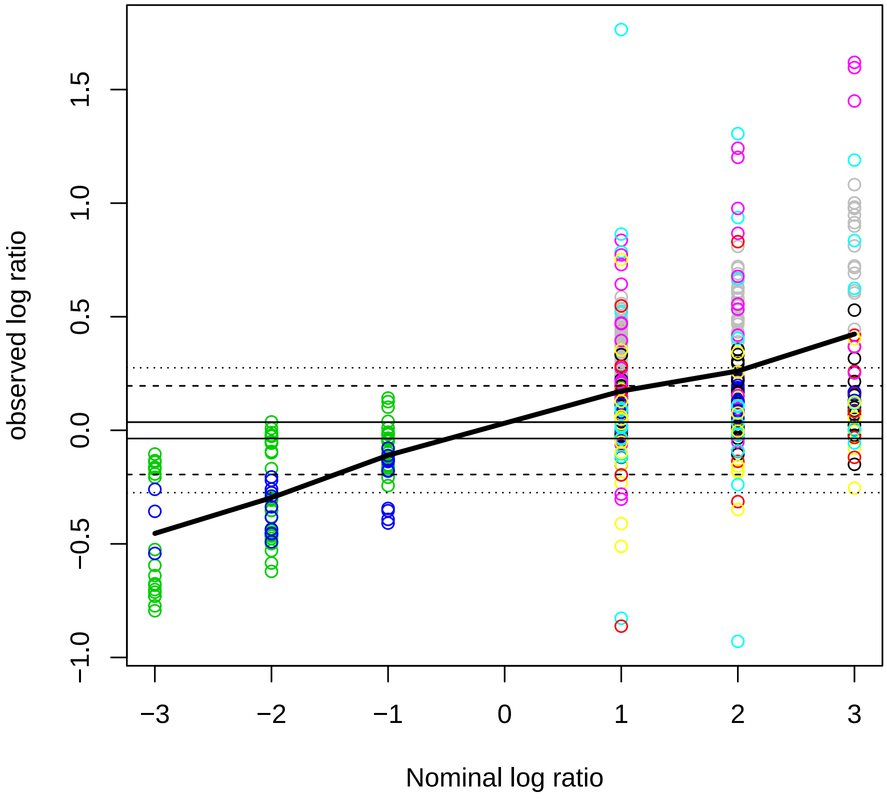

Figure 6b) Like a) but the observed fold changes were calculated for spiked in genes with nominal concentrations no higher than 2pM.
